# The Effect of *Aloe vera* and Chlorhexidine as Disinfectants on the Success of Selective Caries Removal Technique: A Randomized Controlled Trial

**DOI:** 10.1155/2022/9474677

**Published:** 2022-05-04

**Authors:** Ahmad Al-Abdullah, Samer Edris, Amjad Abu Hasna, Lara Steffany de Carvalho, Talal Al-Nahlawi

**Affiliations:** ^1^Department of Operative Dentistry and Endodontics, Faculty of Dentistry, Syrian Private University, Damascus, Syria; ^2^Department of Restorative Dentistry, Endodontics Division, Institute of Science and Technology, São Paulo State University (UNESP), São José Dos Campos, SP, Brazil; ^3^The National Institute of Higher Education and Post-graduation Priest Gervásio (INAPÓS), Pouso Alegre, MG, Brazil

## Abstract

This study aimed to evaluate the effect of *Aloe vera* and chlorhexidine “CHX” as disinfectants on the success of selective caries removal technique in deep carious lesions. A total of 60 patients with: (I) deep class II carious lesion diagnosed with reversible pulpitis; (II) good oral hygiene; (III) no gingival recession or periodontal diseases; (IV) no antibiotic or antifungal treatment in the last three months; and (V) no systematic disease or pregnancy were included in the study. Sixty patients were distributed randomly to three experimental groups (*n* = 20): Group 1: no disinfectant solution was applied (control group); group 2: the cavity was filled with 2% CHX for 5 mins and then dried with a sterilized cotton pellet; group 3: the cavity was filled with *Aloe vera* extract for 5 mins and then dried with sterilized cotton pellet. One week later, only teeth with vital pulp characteristics were restored definitely with resin composite. After 18 months, clinical and radiographic examination was performed by using a blinded separated evaluator. The data were tabulated and analyzed using the chi-square test by SPSS 13.0 with a significance level (*p* ≤ 0.05). It was observed that 13 teeth of the control group, 14 teeth of the CHX group, and 16 teeth of the *Aloe vera* group were diagnosed with healthy vital pulp after 18 months. There was no significant difference between the CHX and the control group; however, there was a significant difference between the *Aloe vera* and control group (*p* ≤ 0.007). *Aloe vera* extract as a cavity disinfectant increases the success rate of selective caries removal technique of deep carious lesions.

## 1. Introduction

Dental caries is a common disease principally caused by bacteria that break down foods and produce acid that destroys dental hard tissues leading to tooth cavities [1]. Consequently, caries can reach the dental pulp and cause reversible or irreversible pulp inflammation (pulpitis) [2].

Its management consists of decayed tissue removal, cavity disinfection, and adequate restoration [3]. However, different approaches may be considered depending on lesion extension, pulp tissue status, patient's age, and other factors [4] in which a selective removal of decayed tissue may be indicated in asymptomatic deep carious lesions [3], combined with antimicrobial agents to arrest caries progression [5].

Chlorhexidine (CHX) is the most frequently used agent to reduce plaque aiming to control caries progression [6], it has antimicrobial action [7] and improves cervical marginal sealing [8]. Besides, *Aloe vera*, an herbal medicine, belongs to the Liliaceae family [9]. Like other herbal extracts [10, 11], it was indicated as an oral antiseptic for the prevention of dental caries and periodontal diseases [12] because of its antimicrobial effect, anti-inflammatory action [13, 14], and bone-dentin bridge formation inducing property [15, 16].

The aim of this study was to evaluate the effect of two cavity disinfectants on the success of the selective caries removal technique of deep carious lesions combined with asymptomatic reversible pulpitis. The null hypothesis was that both disinfectants have no effect on the success of the selective caries removal technique.

## 2. Methodology

### 2.1. Patients' Selection

A total of 60 patients (20–40 years) were included in this randomized controlled trial after approval by the Scientific Research Ethics Committee of the Syrian Private University (*n* 117.06/01/2016). An informed consent form was signed by all the participants of the study.

### 2.2. Inclusion and Exclusion Criteria

Patients with (I) deep class II carious lesion diagnosed with reversible pulpitis, confirmed by a positive response to pulp vitality test (cold test) and absence of any periapical lesions; (II) good oral hygiene; (III) no gingival recession or periodontal diseases; (IV) no antibiotic or antifungal treatment in the last three months; (V) no systematic disease or pregnancy were included in the study. Instead, all patients who did not fulfill any of the previous criteria were excluded, in addition to those presenting teeth that could not be isolated with a rubber dam.

To guarantee the allocation concealment, all patients were numbered randomly from 1 to 60, and a random integer generator (https://www.random.org) was used to assign the patients for the three experimental groups. Patients were informed about the three experimental groups that they will be involved; however, they were not informed about which group specifically they were assigned.

### 2.3. *Aloe vera* Formulation

*Aloe vera* was prepared at the pharmaceutical laboratory of the faculty of pharmacology of the Syrian Private University using mature (two-years-old) and fresh leaves of *Aloe vera* approximately 90–100 cm long after being washed with fresh water. *Aloe vera* thick epidermis was removed and cut transversely into pieces. The solid mucilaginous, thick straw-colored gel was collected in a sterile container. One hundred grams of the gel was mixed in one liter of 2% dimethyl sulfoxide (DMSO) (10%) and kept at 4°C to be used as a stock solution.

### 2.4. Experimental Groups

After local anesthesia with 2% lidocaine 1 : 80000 epinephrine (Dentsply Sirona, York, PA), the isolation with a rubber dam was carried out. The removal of carious tissues was performed using a diamond round bur (Dentsply Sirona, São Paulo, SP, Brazil) with water coolant starting from peripheral walls and extending to gingival walls. Once the pulpal wall was reached, selective removal of caries was performed with caution in which a maximum of 3 × 3 mm-stained soft infected dentin confirmed with caries indicator (Seek, Ultra Dent, USA) were removed [17]. Shortly thereafter, the cavity was washed with 5 ml of distilled water (Prime Dental Products Pvt. Ltd., India) for 10 seconds, and dried using a gentle stream of air and sterilized cotton pellet. Then, the cavity was treated by one of the following protocols (*n* = 20 of each group).

- Group 1: no disinfectant solution was applied (control group).
- Group 2: the cavity was filled with 2% CHX for 5 mins and then dried with a sterilized cotton pellet.
- Group 3: the cavity was filled with *Aloe vera* extract for 5 mins and then dried with a sterilized cotton pellet.

All teeth were then restored temporarily with glass ionomer cement “GIC” (Kavitan plus SpofaDENTAL, Czech Republic) after placement of the Tofflemire matrix. One week later, the vitality test was performed again. Only teeth with vital pulp characteristics proceeded to GIC partial removal and then restored definitely with resin composite restoration obtaining tight contact points. Any teeth with irreversible pulpitis or necrosed pulp were directed to endodontic treatment.

### 2.5. Follow-up

Patients were contacted monthly by phone to control any possible complications. After 18 months, all included patients were recalled to evaluate the pulp vitality or any signs and symptoms of inflammation or infection. Clinical and radiographic examination was performed by a blinded separated evaluator.

The following criteria were used to evaluate the success of the treatment: (I) positive response to cold, heat, and electric pulp vitality test; (II) absence of any radiographic lesions; and (III) absence of pain upon percussion or palpation. Any restored teeth lacking one of the previous criteria was considered as failure and was directed to endodontic treatment.

### 2.6. Statistical Analysis

**Figure 1:**
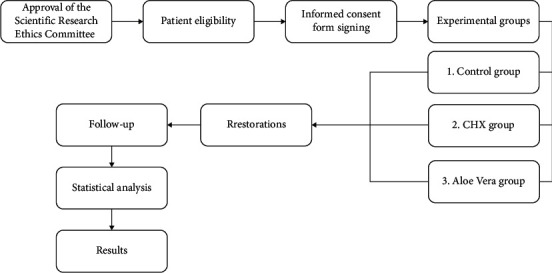
Flowchart of the trial.

Figure 1 is a flowchart that explains the progress of the trial till the statistical analysis, in which the data were tabulated and analyzed using the chi-square test by SPSS 13.0 with a significance level (*p* ≤ 0.05).

## 3. Results

It was observed that 13 teeth of the control group, 14 teeth of the CHX group, and 16 teeth of the *Aloe vera* group were diagnosed with healthy vital pulp after 18 months (Table 1).

There was no significant difference between CHX and control groups; however, there was a significant difference between *Aloe vera* and control groups (*p* ≤ 0.007) as presented in Table 2.

## 4. Discussion

Phytotherapy is gaining a greater space in dentistry because of the antimicrobial effect, anti-inflammatory action, and biocompatibility of diverse herbal medicines [11, 16, 18]. *Aloe vera* extract as an herbal medicine was tested in this study to evaluate its effect on the success of the selective caries removal technique of deep carious lesions combined with asymptomatic reversible pulpitis when used as a cavity disinfectant. The null hypothesis is to be rejected as *Aloe vera* extract presented a significant statistical difference when compared to CHX and the control group (no disinfectant).

This is a clinical trial, and all studies included a risk while performing the treatment. In this study, the informed consent form alerted the patients about a minimal risk of treatment failure and the necessity to perform an endodontic treatment. Both *Aloe vera* and CHX are biocompatible [19, 20]; therefore, there was no risk while using these materials in humans.

In this study, it was proved that *Aloe vera* extract has a positive effect as a cavity disinfectant on the success of selective caries removal technique of deep carious lesions combined with asymptomatic reversible pulpitis when compared to the CHX and control group (no disinfectant). This may be explained by the antimicrobial action of *Aloe vera* reported previously, it is effective over a variety of cariogenic and periodontopathic microorganisms including *Streptococcus mutans* which has a principal role in caries development [12]. This effectivity indicated the use of *Aloe vera* extracts as a remedy for dental caries prevention and as a mouthwash [13] as it minimizes secondary caries and renders a long-term restorative success.

Acemannan, a D-isomer mucopolysaccharide in *Aloe vera* leaves, was also indicated as a pulp-capping material for primary teeth, as it is biocompatible and it enhances reparative dentin formation [21] and for vital pulp therapy including pulpotomy for immature permanent teeth [15]. This may explain the superior results found in *Aloe vera* group.

The selective caries removal technique of deep carious lesions was reported in the literature using air abrasion [22]; however, it may be applied using mechanical burs [23], it presents a higher success in maintaining pulp vitality when compared to other techniques like stepwise, or nonselective removal [24]. In this study, the success rate was greater when the technique was combined with the use of *Aloe vera* as a cavity disinfectant.

CHX is indicated as a cavity disinfectant for atraumatic restorative treatment and selective caries removal technique [25, 26]. However, Walsh et al. found in a systematic review that there is little evidence about the effectivity of CHX in the prevention of caries or the reduction of *S. mutans* in dental cavities [27]. In this study, it was found that CHX has no significant difference when compared to the control group on the selective caries removal technique of deep carious lesions. Finally, the success of deep carious lesions management is affected by diverse factors including the caries removal technique, the disinfectant type, the restoration technique, and the restorative materials [28–30].

## 5. Conclusion

The use of *Aloe vera* extract as a cavity disinfectant increases the success rate of the selective caries removal technique of deep carious lesions.

## Figures and Tables

**Table 1:** Number (and percentage) of failures and successes of all experimental groups.

Experimental group	Failure	Success	Total
Control	7 (35%)	13 (65%)	20 (100%)
CHX	6 (30%)	14 (70%)	20 (100%)
*Aloe vera*	4 (20%)	16 (80%)	20 (100%)
Total	17 (28.3%)	43 (71.7%)	60 (100%)

**Table 2:** Statistical difference between failures and successes after 18 months.

Experimental group	Chi-square	Degree of freedom	*p* value	Significant difference
Control	1.800	1	0.18	No
CHX	3.200	1	0.074	No
*Aloe vera*	7.200	1	0.007	Yes

## Data Availability

The data used to support the findings of this study are available in the Harvard Dataverse repository, https://doi.org/10.7910/DVN/F8BK4G.
